# Structural Characteristics of HLA-DQ that May Impact DM Editing and Susceptibility to Type-1 Diabetes

**DOI:** 10.3389/fimmu.2013.00262

**Published:** 2013-08-29

**Authors:** Zemin Zhou, Peter E. Jensen

**Affiliations:** ^1^ARUP Laboratories, Department of Pathology, University of Utah, Salt Lake City, UT, USA

**Keywords:** type-1 diabetes, HLA-DQ, HLA-DM, invariant chain, autoreactive T cells, negative selection

## Abstract

Autoreactive CD4+ T cells initiate the chronic autoimmune disease Type-1 diabetes (T1D), in which multiple environmental and genetic factors are involved. The association of HLA, especially the DR-DQ loci, with risk for T1D is well documented. However, the molecular mechanisms are poorly understood. In this review, we explore the structural characteristics of HLA-DQ and the role of HLA-DM function as they may contribute to an understanding of autoreactive T cell development in T1D.

## Introduction

Multiple factors contribute to the chronic autoimmune disease type-1 diabetes (T1D) characterized by selective destruction of pancreatic β cells. To complement β cell deficiency, life-long insulin replacement is required to maintain glucose metabolism. There is evidence that both genetic and environmental factors contribute to the etiology of T1D. Genome wide association analysis data indicate that the highly polymorphic major histocompatibility complex (MHC), including both MHC class I and class II (MHCI and MHCII), contributes approximately 50% of genetic susceptibility to T1D (1). Individuals with MHCII DR3-DQ2 and DR4-DQ8 haplotypes have a significantly higher risk of T1D and DQ6 (DQA1^∗^0102/DQB1^∗^0602) is dominantly protective in Caucasians, Mexicans, and other Latin American populations (1–3). A number of studies have demonstrated the peptide-binding specificity of DQ8 as well as T cells from T1D that recognize pancreatic autoantigens presented by DQ8 (4–8). Compared with the DQ2 and DQ8 homozygous individuals, DR3-DQ2/DR4-DQ8 heterozygotes (DRB1^∗^0301-DQA1^∗^0501-DQB1^∗^0201/DRB1^∗^04-DQA1^∗^0301-DQB1^∗^0302) have the highest risk in whites of European and Northern African decent (9). Haplotype sharing analysis in siblings also shows that the risk for T1D is dramatically increased in DR3/4-DQ2/8 siblings (10). Another study of 607 Caucasian families and 38 Asian families further confirmed the association of DQ2 and DQ8, especially the trans-dimer DQ2-8, with the highest risk of T1D (11). These striking observations raise several open questions: (a) what structural features distinguish DQ molecules associated with risk for T1D; (b) why do heterozygotes have even greater risk for T1D than individuals homozygous for DQ2 or DQ8; (c) how do the autoreactive CD4+ T cells that mediate β cell destruction develop and escape negative selection in the thymus. In this review, we will focus on the function of MHCII molecules and their role in selection of autoreactive CD4+ T cells.

## MHCII Function in Antigen Presentation

In the adaptive immune system, MHCI and MHCII molecules play critical roles by presenting peptides on the surface of antigen presentation cells (APC) to select or activate CD8+ and CD4+ T cells, respectively (12). MHCI and MHCII share very similar structure in the peptide-binding groove and both can load with endogenous or exogenous peptides through two sets of non-covalent interactions: sequence dependent anchor-pocket interactions and conserved hydrogen-bond networks formed between the peptide and non-polymorphic amino acids in MHC. However, the peptide-binding groove of MHCII is open in both sides, compared with the closed binding site in MHCI; therefore, MHCII can present relatively longer peptides. Extra residues in the *N*-terminus of the bound peptide, such as P-1 and P-2, are important for the stability of MHCII/peptide complexes (13). MHCII molecules initially assemble with invariant chain (Ii) in the endoplasmic reticulum (ER) and the peptide-binding groove is occupied by a disordered region of Ii to prevent the loading of other ligands in the ER. After translocation into late endosomal compartments, Ii is processed by endosomal proteases and a segment of Ii, CLIP (class II-associated Ii peptide), occupies the peptide-binding groove. The dissociation of CLIP from the peptide-binding groove is necessary for the loading of other peptides, which is accelerated by a non-classical MHC class II molecule, HLA-DM (DM) (14). DM can catalyze multiple subsequent rounds of peptide exchange, editing the repertoire of presented peptides, and favoring the most stable peptide complexes.

## Molecular Mechanism of DM-Mediated Peptide Editing and Its Potential Role in T1D

**Figure 1:**
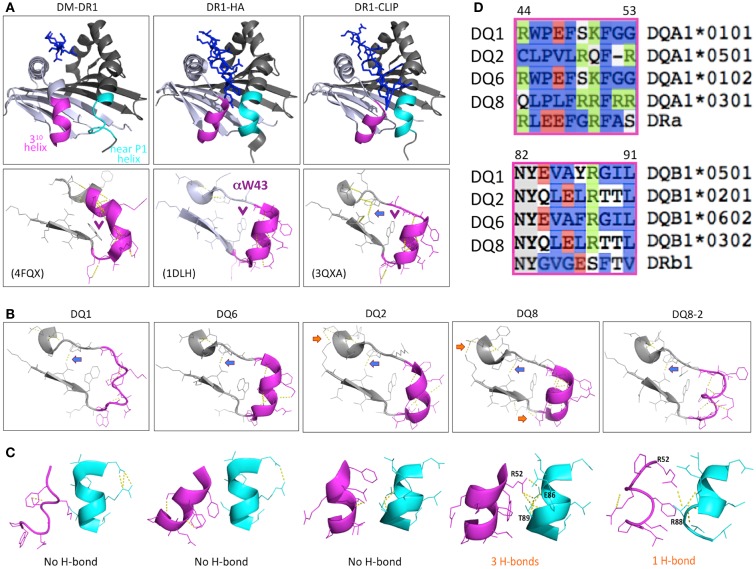
**Structure characteristics of DR1 and T1D sensitive, neutral, and protective DQ molecules**. **(A)** The structure of DR1 showing with P1 pocket empty (left of upper panel, in co-structure of DM-DR1), bound with high affinity HA peptide (middle), and bound with low affinity CLIP (right). The purple and cyan colors show the conformational difference of the two helices near the P1 pocket of the DR1 peptide-binding groove in the crystal structures. The lower panel shows the H-bond between 3^10^ helix and β-sheet, and the αW43 position (purple arrow “ →”). The unique H-bond in DR1-CLIP is showed by blue arrow “ →”. **(B)** Conformational difference of the 3^10^ helix, β-sheet, and inter-helix H-bond(s) in different DQ molecules. There is a conserved H-bond formed in all of the DQ molecules and DR1 bound with CLIP peptide (blue arrow “ →”), indicating a similar status among these molecules. Also, extra H-bond(s) are found in T1D-associated DQ2 and DQ8 (orange arrow “ →”), suggesting a stabilized conformation in this region, compared with DQ1 or DQ6. **(C)** Conformational differences in the α chain 3^10^ helix, the β chain near the P1 helix, and the H-bond(s) interactions between the two helices. DQ8 have 3 H-bonds formed between the two helices, and DQ8-2 has 1 H-bond, compared with DQ1, DQ2, and DQ6, with no H-bonds. **(D)** Sequence comparison of different DQ molecules and DR1 in the helix regions.

The general function of DM is well defined but many questions have remained about its precise mechanism of action (14). The possibility that DM selectively disrupts conserved hydrogen bonds between peptide and MHCII had been proposed as a potential mechanism (15, 16); however, subsequent analysis of substituted MHCII molecules with disrupted H-bonds ruled out this mechanism in its simplest form (17, 18). It has been suggested that the interaction of DM with MHCII activates the empty or inactive form of MHCII to be active for peptide loading (19, 20). MHCII molecules with an empty P1 pocket can associate with DM while the filled form has been reported to interact poorly with DM (21). Molecular dynamics simulation studies indicated that the peptide-binding groove in the bound, partially filled, or empty states are significantly different (22–24), indicating that the interaction of DM and MHCII might induce a conformational rearrangement of peptide-binding groove, especially the α53–65 region around P1 pocket of MHCII (19). Recent advances with the co-crystallization of DM and DR (25), and the co-crystallization of DM and DO (26), another non-classical MHCII that inhibits DM function (14), provide a significant advance in our understanding of the interaction of DM with MHCII, confirming that DM binding is associated with a major structural rearrangement of the MHCII α53–65 region (Figure 1A) that precludes occupancy of the region of the peptide-binding groove that normally accommodates the peptide *N*-terminus, including the P1 anchor residue.

Genetic studies of the limited polymorphisms of DMα and DMβ in different populations indicate that specific DM alleles are associated with T1D (27–29). Interestingly, patients with T1D show relatively high levels of CLIP on the surface of lymphocytes (30), and T1D-like NOD mice also display high CLIP levels (31), indicating that DM is inefficient in removing CLIP from specific MHCII molecules expressed in individuals with T1D and NOD mice. A natural deletion of arginine in α53 of DQ2 has been demonstrated to reduce affinity for DM, explaining inefficient DM-mediated peptide exchange in T1D-associated DQ2 molecules (32, 33), further supporting the idea that inefficient DM editing may play a critical role in T1D-associated autoreactive CD4+ T cell development (32, 34). The coincidence of high CLIP expression might be a general indicator of poor DM editing function with T1D-associated DQ molecules, and it is also plausible that high levels of CLIP select CD4 T cells are cross-reactive and autoreactive. Interestingly, Ii deficient NOD mice are protected from T1D (35), providing further evidence for the potential role of CLIP in autoreactive T cell development; however, there is no direct evidence currently supporting this hypothesis.

## Structural Characteristics of T1D-Associated DQ Molecules

The structure of the T1D sensitive, neutral, and protective DQ molecules, including DQ2 (PDB ID: 1S9V) (36), DQ8 (1JK8, 2NNA, and 4GG6) (37–39), DQ8-2 (4D8P) (40), DQ1 (3PL6) (41), and DQ6 (1UVQ) (42), have been recently solved. These DQ molecules share the general structural characteristics of MHCII with an open peptide-binding groove interacting with variable length peptides through a nine-residue binding “core”. In the core, preferred amino acids anchor the peptide at positions 1, 4, 6, 7, and 9 (32). However, the conformations of the 3^10^-helix region (43), which is in the DM-MHCII contact surface (25) and affects the sensitivity of DM-MHCII interaction (43), are apparently variable among the different DQ structures (Figures 1A,B). Interestingly, there are 3 H-bonds formed between the two helices in the α and β chains of DQ8 and 1 H-bond in DQ8-2, but no H-bond in the low T1D risk DQ1 or DQ6 molecules (Figure 1C). The conformation of the two helices and the number of inter-helix H-bonds in DQ8 are not dependent on the sequence specificity of bound peptide (37–39). Sequence comparison of the helical regions of the α and β chains among these DQ molecules shows that, in T1D-associated DQ2 and DQ8, the 3^10^ helix of the α chain includes several positively charged residues and the helix of the β chain has some negatively charged or uncharged hydrophilic residues with the potential to form H-bond(s); while in DQ1 and DQ6, those residues are hydrophobic (Figure 1D). The structure differences between DQ8 and other DQ molecules indicates that H-bond(s) might play a role in regulation of the sensitivity to DM editing by further stabilizing the DM contact region, providing an energetic barrier to formation of the DM-bound conformation. The structural differences between DQ8 and DQ2 suggest that different mechanisms might be responsible for the relative inefficiency of DM-mediated peptide editing in these molecules (33). The sensitivity of the T1D-associated DQ8 and DQ8-2 molecules to DM editing, and the potential inter-helix H-bond(s) or other structural features that might impact DM catalytic potency warrant further investigation.

It is still unclear why heterozygosity for DQ2/8 confers exceptionally high risk for T1D. APC in individuals with this haplotype co-express four distinct DQ molecules, including the trans-encoded DQ2-8 and DQ8-2 mixed haplotype molecules and the parental DQ2 and DQ8 proteins. Peptides eluted from the 293T cells expressing different DQ molecules show that the peptide-binding motifs of these DQ molecules are unique (8), supporting the hypothesis that the trans-dimers in heterozygotes might confer risk through independent presentation of specific self-peptides (44, 45). However, it is also possible that the higher risk of DQ2/8 heterozygous is due to an expanded repertoire of presented self-peptides by the combination of four DQ molecules. A study comparing gluten-specific T cells from Celiac disease patients demonstrated the potential for T cells to cross-react with DQ8 and the DQ2-8 trans-dimer (46), raising the possibility that T cell cross-reactivity might somehow contribute to the etiology of autoimmunity associated with DQ2/8 heterozygosity. Further studies are needed to explore these various possibilities.

## The Development of T1D-Associated Autoreactive T Cells

A big challenge in this field is to understand how autoreactive T cells develop, survive negative selection, and become activated to mediate tissue damage. In the thymus, the autoimmune regulator (Aire) regulates the ectopic expression of “tissue-restricted” antigens in medullary thymic epithelial cells (mTECs). The fate of thymocytes is determined by the affinity of expressed T cell receptor (TCR) for self-peptide-MHC complexes (47). Theoretically, the T cell precursors that bind strongly to self-peptide-MHC complex on thymic dendritic cells (DCs) and mTECs will be deleted, and all remaining mature T cells are self-tolerant. However, the identification of autoreactive T cells in T1D patients, and even in healthy subjects, indicates that negative selection in the thymus is incomplete (48). Several mechanisms have been proposed for inefficient deletion of autoreactive T cells in the thymus, including differences in autoantigen expression in the thymus and periphery, autoantigen posttranslational and posttranscriptional modification, autoantigen polymorphisms (49), and mechanisms through which key self-peptides can be presented on the cell surface through alternative pathways (34), or as a result of poor DM editing function (32). In addition, T cell cross-reactivity between microbial and self-antigens may also play an important role in the development of autoimmunity (50).

**Figure 2:**
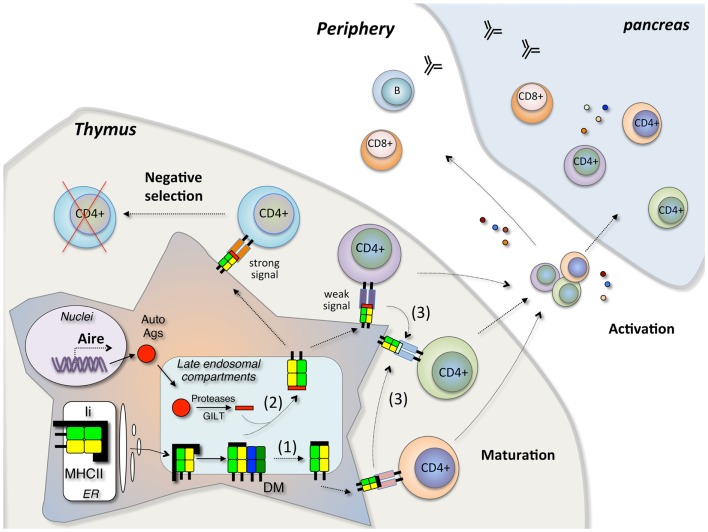
**Model of autoreactive CD4+ T cell development in T1D**. In the thymus, Aire regulates tissue-specific autoantigen expression. Autoantigen peptides are processed in the late endosomal compartment and loaded in the peptide-binding groove of MHCII by DM editing. In case of inefficient DM editing, the pre-bound CLIP peptide may escape peptide exchange, resulting high levels of CLIP presentation (1). Secondly, the inefficient DM editing may lead to presentation of both low affinity and high affinity peptides on the cell surface (2). The stable MHCII-peptide complex will deliver strong signal through the T cell receptor (TCR) and induce the deletion of CD4+ T cells by negative selection, while the unstable MHCII-peptide complex will deliver weak signal and this signal may induce the positive selection of CD4+ T cells. Alternatively, the unstable complexes presented on the cell surface may be more susceptible to DM-independent peptide exchange (3). Those escaped CD4+ T cells will migrate into the periphery and initiate the β cell destruction in pancreas under certain conditions.

Based on current findings, we postulate that the T1D-associated DQ molecules (DQ2, DQ8, and the DQ2/8 trans-dimers) share a common feature, a relative resistance to DM-mediated peptide exchange, and editing. This impacts antigen presentation in two ways (Figure 2). A substantially increased fraction of MHCII molecules escape even one round of peptide exchange, resulting in high levels of CLIP presentation in the periphery and presumably also in the thymus. Secondly, a reduction in the efficiency of further peptide editing may lead to presentation of an array of relatively unstable peptide complexes. High levels of CLIP in the thymus might result in positive selection of T cells that cross-reactive with autoantigens in the periphery, or a reduction in the negative selection of self-reactive T cells, as is seen in the extreme case in mice with targeted deletion of DM (51). Increased presentation of unstable self-peptide complexes might also lead to inefficient negative selection and survival of T cells with a capacity to be activated in the periphery under conditions where the concentration of pancreatic β cell antigens is high. Alternatively, unstable complexes may be more susceptible to DM-independent peptide exchange in the periphery, promoting the activation of “type B” T cells that recognize β cell peptides bound to MHCII through an alternative register or conformation generated through alternative presentation pathways (34). These potential mechanisms may contribute to the pathogenesis of T1D but further elements are needed to explain the specificity for β cells as opposed to other tissues. This is presumably related to the capacity of the T1D-associated DQ molecules to bind and present key β cell self-peptides.

## Conclusion

Type-1 diabetes is a chronic autoimmune disease affected by both environmental and genetic factors. The mechanism(s) responsible for the high genetic risk associated with HLA genotype, and especially DQ2, DQ8, and DQ2/8 heterozygosity, remains poorly understood despite the obvious role of these molecules in antigen presentation. Reduced DM editing of T1D-associated DQ-peptide complexes combined with T cell cross-reactivity may contribute. Further analysis of structural and functional characteristics that distinguish disease-associated DQ molecules from neutral or protective alleles is likely to provide insights into the fundamental question of why HLA haplotype is such an important factor in determining risk for T1D.

## Conflict of Interest Statement

The authors declare that the research was conducted in the absence of any commercial or financial relationships that could be construed as a potential conflict of interest.
